# The impact of interventions for the primary prevention of hypertension in Sub-Saharan Africa: A systematic review and meta-analysis

**DOI:** 10.1371/journal.pone.0219623

**Published:** 2019-07-19

**Authors:** Akosua A. Wamba, Noah F. Takah, Cathy Johnman

**Affiliations:** 1 Emergency Department, Korle-Bu Teaching Hospital, Accra, Ghana; 2 Clinical Research Department, Faculty of Infectious and Tropical Diseases, London School of Hygiene and Tropical Medicine, London, United Kingdom; 3 Institute of Health and Well-being, University of Glasgow, Glasgow, United Kingdom; University of Mississippi Medical Center, UNITED STATES

## Abstract

**Background:**

The prevalence of hypertension is highest in the African Region with 46% of adults aged 25 and above diagnosed with hypertension, while the lowest prevalence of 35% is found in the Americas. There is sparse evidence on the approaches used to prevent hypertension in Sub-Saharan Africa and the effectiveness of these approaches. It is therefore imperative that a systematic review; which synthesises all the available evidence on the approaches and their impact is conducted to inform public health policy and practice.

**Objective:**

To synthesise evidence on the interventions used for the primary prevention of hypertension in Sub-Saharan Africa and to evaluate the effectiveness of these interventions in reducing blood pressure, hypertension prevalence and the risk factors for hypertension.

**Methods and results:**

This systematic review was reported per the Preferred Reporting Items for Systematic Reviews and Meta-Analysis (PRISMA) guidelines. Bibliographic databases were searched on the 4^th^-17^th^ of January 2018 from 1970 to January 2018 and on the 5^th^ of May 2019 from 1970 to May 2019, for studies focusing on the primary prevention of hypertension in communities in Sub-Saharan Africa. A narrative synthesis was conducted based on study interventions and outcomes. Also, a meta-analysis was carried out using pooled mean differences; using a random effects model of generic inverse variance option in RevMan. A total of 854 studies were identified after deduplication, with thirteen studies meeting the inclusion criteria. Six studies with varying interventions and methodologies observed a significant pooled reduction in systolic blood pressure of -3.3mmHg (95%CI -4.64 to -1.96) and a reduction of -2.26mmHg (95%CI -6.36 to 1.85) in diastolic blood pressure, which was not statistically significant (p = 0.28). Also, moderate to significant heterogeneity was observed (I^2^ = 68% and 99%) for the systolic and diastolic blood pressure respectively. Intervention and study design accounted for 100% heterogeneity for both systolic and diastolic blood pressure (r^2^ = 100%).

**Conclusion:**

Health promotion and interventions targeting various risk factors of hypertension and, salt consumption restriction interventions have been employed in Sub-Saharan Africa with varying levels of success. We recommend that higher quality studies and a meta-analysis are needed to evaluate the impact of these interventions and to inform public health policy and practice.

## Introduction

The WHO STEPS survey conducted between 2003 and 2009 in 20 African countries reported high rates of hypertension, with prevalence by sex ranging from 15% to 45% and higher rates of hypertension observed in men compared to women [1]. Similarly, another study estimated the prevalence of hypertension to range from 19.3% in Eritrea to 39.6% in Seychelles [2]. A systematic review [3] was unable to estimate the overall prevalence due to high study heterogeneity but found that hypertension detection was low and ranged from 11% to 44%. Hypertension was also found to be higher in urban areas compared to rural areas [3]. Despite the progress that has been made in recent years in the prevention of hypertension globally, it still is an important public health challenge especially in SSA due to an increase in unhealthy behaviours, poor health systems, and urbanisation [4]. Many studies have been published on the prevalence, detection, and control of hypertension in SSA, but little is known about the status of hypertension prevention in the region. Moreover, sparse evidence is available for informed policy decisions [5].

Several systematic reviews and meta-analyses evaluating the effect of specific interventions have been conducted, but none of them has evaluated all the interventions for the primary prevention of hypertension employed in SSA. For instance, a systematic review and meta-analysis [6] of 24 RCTs involving 23,858 participants, on the effects of dietary interventions on blood pressure calculated a pooled overall reduction of -3.07mmHg (95% CI -3.85 to -2.30) in SBP and -1.81 (95% CI -2.24 to -1.38) in DBP. However, the study did not include trials conducted in SSA and only focused on dietary intervention trials. Similarly, another systematic review [7] evaluated the effect of behavioural counselling interventions promoting physical activity on CVD. They estimated that high-intensity dietary counselling, with or without, physical activity resulted in a -1.5 (95% CI -0.9 to -2.1) decrease in SBP and a -0.7 (95% CI -0.6 to -0.9) decrease in SBP. The study is limited because only 13 out of 102 RCTs were judged to be of good quality. Also, many of the reviewed trials had high attrition rates and short follow–up periods making the study susceptible to selection bias, attrition bias and systematic errors [7]. Furthermore, a Cochrane review [8] on population-level interventions in government jurisdictions for dietary sodium reduction identified 15 national initiatives, including more than 260,000 people. However, the study only included high and upper- middle income countries. Also, a pooled estimate could not be calculated due to high heterogeneity (I^2^ > 90%).

It must be acknowledged that two systematic reviews conducted in SSA were identified [9, 10]. However, one of them focused only on salt reduction interventions implemented in SSA, with most of the studies targeting hypertensive individuals [9]; making it difficult to evaluate the effect of the interventions on the primary prevention of hypertension. Also, the second systematic review [10] focused on the prevention of ischaemic heart disease but did not include important interventions relevant for hypertension prevention such as sodium restriction. Furthermore, it included individual-level interventions, the search was limited to 2015 and it is unclear if they had language restrictions. Also, a meta-analysis could not be conducted due to heterogeneity [10]. Lastly, it has been reported that some interventions for the primary prevention of hypertension; which have been successful in higher income countries, may be less effective in Lower Middle-income countries (LMIC). Socio-cultural differences, poor access to health care, rural/urban disparities, a high burden of communicable diseases, inadequate staff numbers and lower per capita income have been implicated, which may hamper the implementation of interventions [9, 11–15].

Therefore, a systematic review and meta-analysis assessing all available hypertension primary prevention interventions implemented in SSA, and measuring their effectiveness in reducing blood pressure, hypertension prevalence or key risk factors of hypertension, is necessary to inform policy and public health practice in the region.

## Methods

This systematic review was performed and reported per the Preferred Reporting Items for systematic review and meta-analysis protocols (PRISMA-P) guideline and checklist [16], see S1 Checklist. Also, the study has been registered, with PROSPERO—registration number CRD42018091422.

We performed a search in the Ovid Medline, Ovid Embase, Cochrane and Web of Science databases from January 1970 to May 2019. The search was conducted from the 4^th^ to the 17^th^ of January 2018 and on the 5^th^ of May 2019, using comprehensive search terms containing relevant keywords and mesh terms available as supplementary documents (S1 and S2 Appendices). References and grey literature were hand searched for relevant articles and authors contacted for additional information where necessary.

Our inclusion criteria included studies conducted in communities, whole or high-risk populations of Sub-Saharan Africa, irrespective of language of publication and study design. Any intervention recommended for the primary prevention of hypertension or targeting the risk factors of hypertension, as contained in widely used guidelines was included in the study. Also, we included studies with blood pressure, hypertension prevalence and urinary sodium excretion outcome measurements.

Studies which were not conducted in at least one Sub-Saharan African country or conducted on solely hypertensive patients in health facilities were excluded, since our focus was on the primary prevention of hypertension. In addition, we excluded conference abstracts, editorials and correspondence as it was unlikely, they held enough information relevant to the study. It has been reported that less than half of all studies, and about 60% of RCTs, initially presented as summaries or abstracts at conferences are subsequently published as peer-reviewed journal articles [17]. Systematic reviews were not included in the study, but relevant studies included in the reviews were extracted and assessed. Finally, studies that did not report outcomes were excluded from the study since they did not have enough information for evaluation. Selection of articles for inclusion was carried out by two researchers. Disagreements on article selection were settled after discussion between A.W and T.N. However, C.J was consulted if an agreement could not be reached between A.W and T.N. The level of agreement between the two researchers, kappa, was calculated using Excel and was found to be 0.76, which shows a substantial agreement between researchers [18].

### Data extraction

The extraction form was developed using forms used by similar systematic reviews as a reference and had the following headings: author, year, study design, study setting, sample size, intervention, blinding, follow-up period, the control group, population under study and study outcomes. The study outcomes mentioned in the eligibility criteria were eligible for extraction. When two blood pressure readings were reported for two separate devices, an average of the readings was extracted. Also, mean differences of continuous variables were retrieved with or without a measure of dispersion, such as standard deviation or confidence intervals. One reviewer extracted data which was cross-checked by another researcher.

### Quality assessment

The Cochrane tool for assessing risk of bias [19] was used to critically appraise the randomised controlled trials while the National Heart Lung and Blood Institute (NHLBI) tool was used to assess observational and quasi-experimental studies [20], see S1 and S2 Files. The risk of bias was assessed at the study level, and all studies were included irrespective of study quality due to the limited number of studies retrieved from the search.

### Data analysis

A meta-analysis was conducted on six articles deemed homogenous enough to be included in the analysis. Only blood pressure mean difference effect sizes were used, also, systolic and diastolic blood pressure was analysed separately. For each trial, we also calculated the variance of the treatment effect for outcomes. We used funnel plots and Egger’s test [21] to detect the presence of publication bias. The analysis was conducted using the random effects model of the generic inverse variance method (GIVM) available in the review manager (RevMan) [22]. This method was chosen because effect sizes for treatment and control groups were reported jointly instead of separately. Heterogeneity amongst studies was measured using the I^2^ statistic, which does not inherently depend upon the number of studies considered, with values of 25%, 50%, and 75% taken to indicate low, moderate, and high levels of heterogeneity, respectively.

Potential sources of heterogeneity were further investigated by meta-regression using Robumeta package in R [23], with moderators suspected to be responsible for heterogeneity in the model explored. The analysis was weighted by the inverse variance of the effect size and the likelihood measures method employed.

## Results

### Study selection

**Fig 1:**
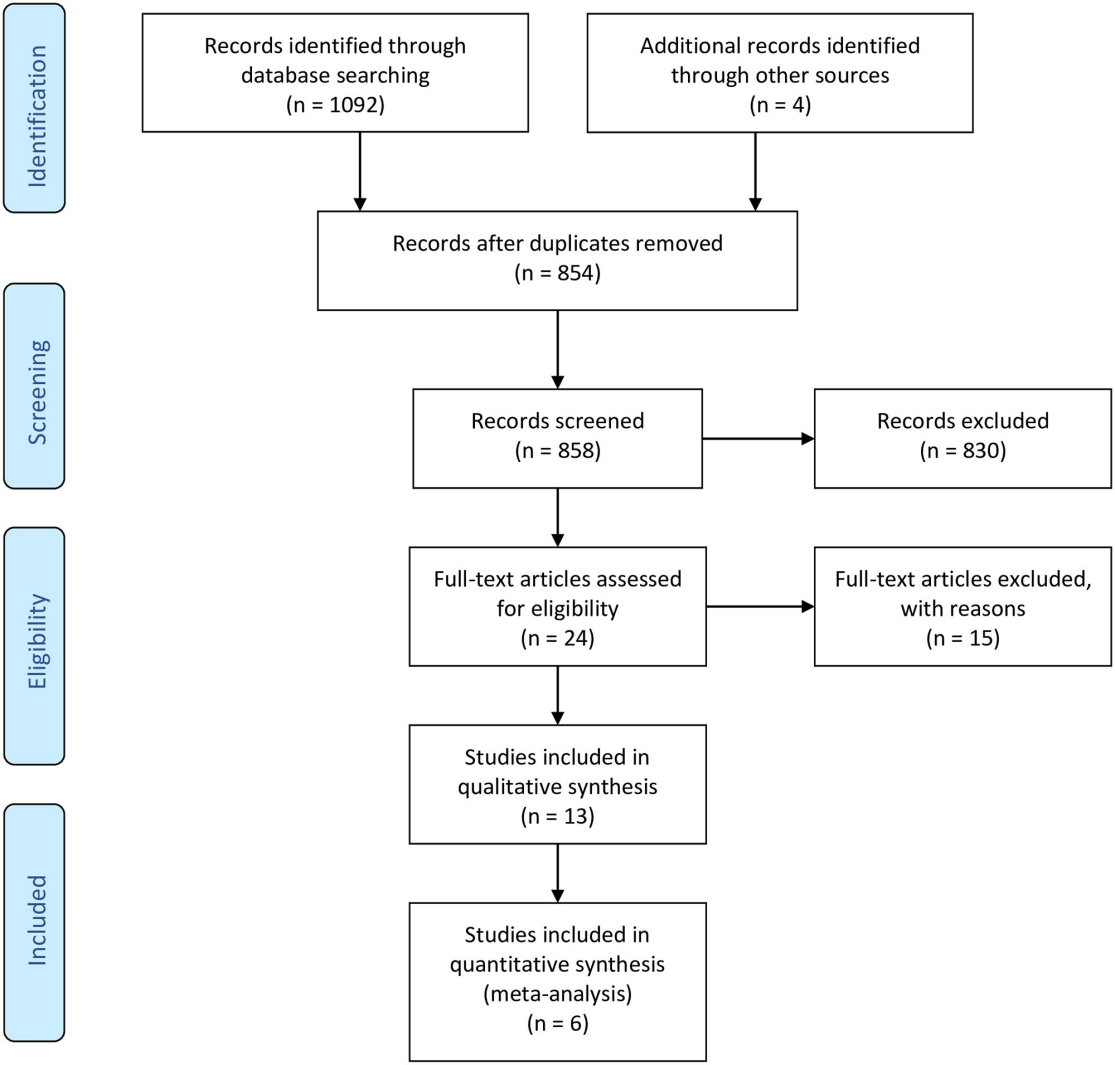
PRISMA study selection flow diagram [16].

Fig 1 shows the number of studies assessed and excluded through the stages of our review and meta-analysis. A total of 13 papers met our inclusion criteria and were included in the review. However, only six papers were deemed homogenous enough to be included in the meta-analysis.

### Study characteristics

The articles reviewed included, two RCTs, five quasi-experimental studies and six cross-sectional studies, with follow-up periods ranging from 5 days to 5.5 years. In addition, sample sizes ranged from 30 to 559,834 individuals, representing six countries namely: Ghana, Nigeria, South-Africa, Kenya, Tanzania and Mauritius. The interventions employed included, salt restriction or limitation, health promotion, physical activity and healthy food price reduction. Also, all the studies involved whole populations or communities except for two studies which targeted high-risk individuals, see Table 1.

**Table 1:** Study characteristics.

Number	First Author	Year	Study design 1	Design 2	Sample size	Intervention	Blinding	Follow-up	Control group	Country
**1**	Ruopeng et al [24]	2013	Cross-sectional	Longitudinal	559,834	Food price reduction	NA	2 years	NO	South-Africa
**2**	Forrester et al. [25]	2005	Randomised trial	Cross-over	58	Salt reduction	NO	4 weeks	YES	Nigeria
**3**	Cappuccio et al [26]	2006	Randomised trial	Cluster	1013	Salt reduction	YES	3,6 months	Yes	Ghana
**4**	Rossouw et al [27]	1993	Quasi experimental	NA	6793	Health promotion (HP)	NO	4years	YES	South-Africa
**5**	Dowse et al [28]	1995	Cross-sectional	Repeat cross-sectional	6381	HP	NO	5years	NO	Mauritius
**6**	Tagoe et al [29]	2011	Cross-section al	Repeat cross-sectional	13357	HP	NO	5 years	NO	Ghana
**7**	Wentzel-Viljoen et al [30]	2017	Cross-sectional	Longitudinal	550	HP	NO	1 year	NO	South-Africa
**8**	Adeyemo et al [31]	2015	Quasi experimental	NA	82	Salt reduction	NO	2 weeks	NO	Nigeria
**9**	Marfo et al [32]	2016	Quasi experimental	NA	170	HP/screening	NO	6months	NO	Ghana
**10**	Van de Vijver et al [33]	2016	Cross-sectional	Repeat cross-sectional	3,220	HP	NO	1.6years	YES	Kenya
**11**	Dickie [34]	2014	Cross-sectional	Longitudinal	240	Physical activity	NO	5.5years	YES	South-Africa
**12**	Pouane [35]	2006	Quasi experimental	NA	76	HP	NO	2 years	NO	South-Africa
**13**	Mtabaji [36]	1990	Quasi experimental	NA	30	Salt reduction	NO	5 days	NO	Tanzania

The RCT papers were observed to have a moderate risk of bias while three observational studies were judged to be fair quality, two of good quality and one of poor quality. In addition, two of the quasi-experimental studies were of good quality, while two were of poor quality and one was fair (see S1 File, Risk of bias observational studies, and S2 File, Risk of bias RCT).

#### Effect of interventions aimed at limiting salt intake on blood pressure

**Fig 2:**
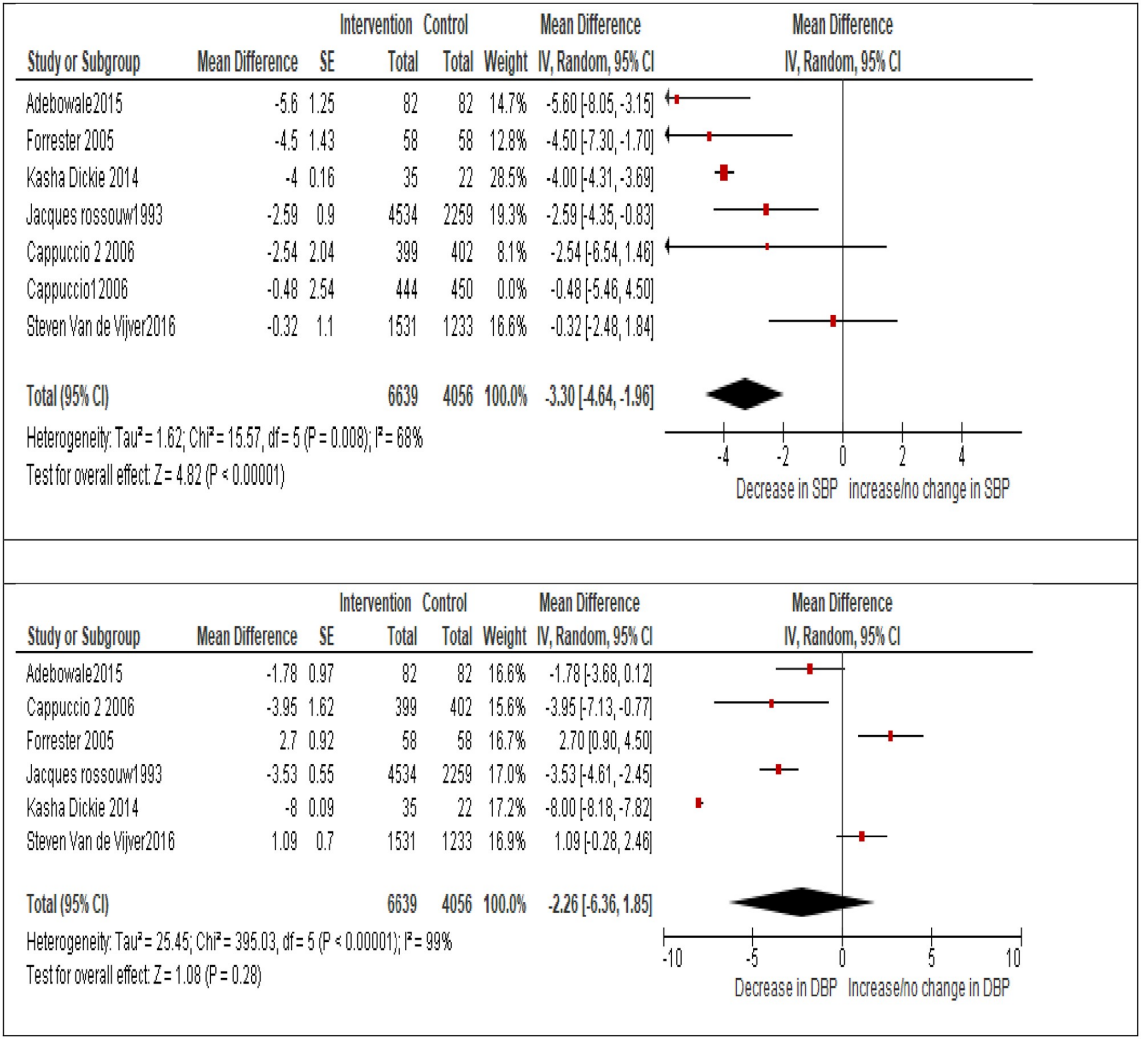
Forest plot showing the effect of study interventions on systolic and diastolic blood pressure.

*Wentzel-Viljoen et al* [37] evaluated a mass media campaign aimed at reducing discretionary salt intake in South-Africa after 1 year and observed a significant increase in the proportion of participants who had taken steps to reduce dietary salt consumption (p<0.001). On the other hand, two studies carried out cross-over RCTs limiting salt intake and observed significant reductions in mean SBP. Similarly, a quasi-experimental study recorded a significant reduction in mean arterial pressure (MAP) during the low salt phase, while a cluster RCT by *Cappuccio et al* [26] found a significant reduction in DBP in the intervention group compared to the control group at six months but observed no significant change in blood pressure at three months (as seen in Fig 2).

#### Effect of health promotion interventions on blood pressure

Two papers [27, 38] evaluated the use of health promotion using mass media campaigns in addition to health education and counselling for high-risk individuals with conflicting results. One of them observed a significant decrease in SBP and DBP four years after the campaign, while the other found no significant change in both SBP and DBP 18 months after the intervention. However, a similar study conducted in Mauritius [28] observed a decrease in hypertension prevalence in both sexes after five years, hypertension prevalence in men, -2.9%mmHg (95% CI -4.7%mmHg to -1.1%mmHg), hypertension prevalence in women, -1.5%mmHg (95%CI -3%mmHg to 0%mmHg).

#### Effect of physical activity on blood pressure

*Dickie et al* [34] measured the difference in blood pressure between physically active and inactive groups at baseline and after five years. They observed a significant reduction in diastolic blood pressure in the active group compared to the inactive group. Similarly, *Pouane et al* [35] evaluated the effect of a health club on blood pressure and found an increase in mean SBP. However, it should be noted that the study was of poor quality.

#### Effect of health promotion on risk factors of hypertension

One study compared two different national surveys conducted five years apart [29], to assess the impact of a mass media, health promotion campaign. However, the study may be underpowered because of a small joint sample size of 13,357, which appears too small for two national surveys in a country with a population of 28 million. Nevertheless, the study reported an increase in the proportion of respondents consuming 1–4 servings of fruit and vegetables daily but noted a decrease in the number of participants consuming more than five servings of fruit and vegetables daily, within the study period. In comparison, the second study [32] was a health promotion study using community pharmacies and targeted high-risk individuals as previously mentioned. There was a significant decrease in the number of obese participants (p = 0.008). In addition, physical activity increased (p = 0.012), salt intake also decreased (0.002). However, only 28 participants in this category were evaluated. Moreover, salt intake was self-reported as opposed to a validated questionnaire or urine sodium excretion which are more accurate and reliable measures of salt consumption.

Only one study investigated the effect of food price reduction on healthy eating [24]. Repeated surveys of about 350,000 participants who had received 10% or 25% discounts were carried out. A 10% and 25% discount on healthy food purchases was associated with an increase in daily fruits and vegetable consumption by 0.38 (95% CI: 0.37–0.39) and 0.64 (95% CI: 0.62–0.65) servings, respectively. Also, individuals receiving a 10% and 25% discount were more likely to have three or more servings of wholegrain foods on a daily basis with an odds ratio (OR) of 2.05 (95% CI: 1.97–2.13) and 2.96 (95% CI: 2.84–3.08) respectively, but less likely to regularly have foods high in sugar with an OR of 0.73 (95% CI: 0.69–0.76) and 0.35 (95% CI: 0.34–0.37), foods high in salt with an OR of 0.59 (95% CI: 0.55–0.62) and 0.26 (95% CI: 0.25–0.28).

### Meta-analysis

**Fig 3:**
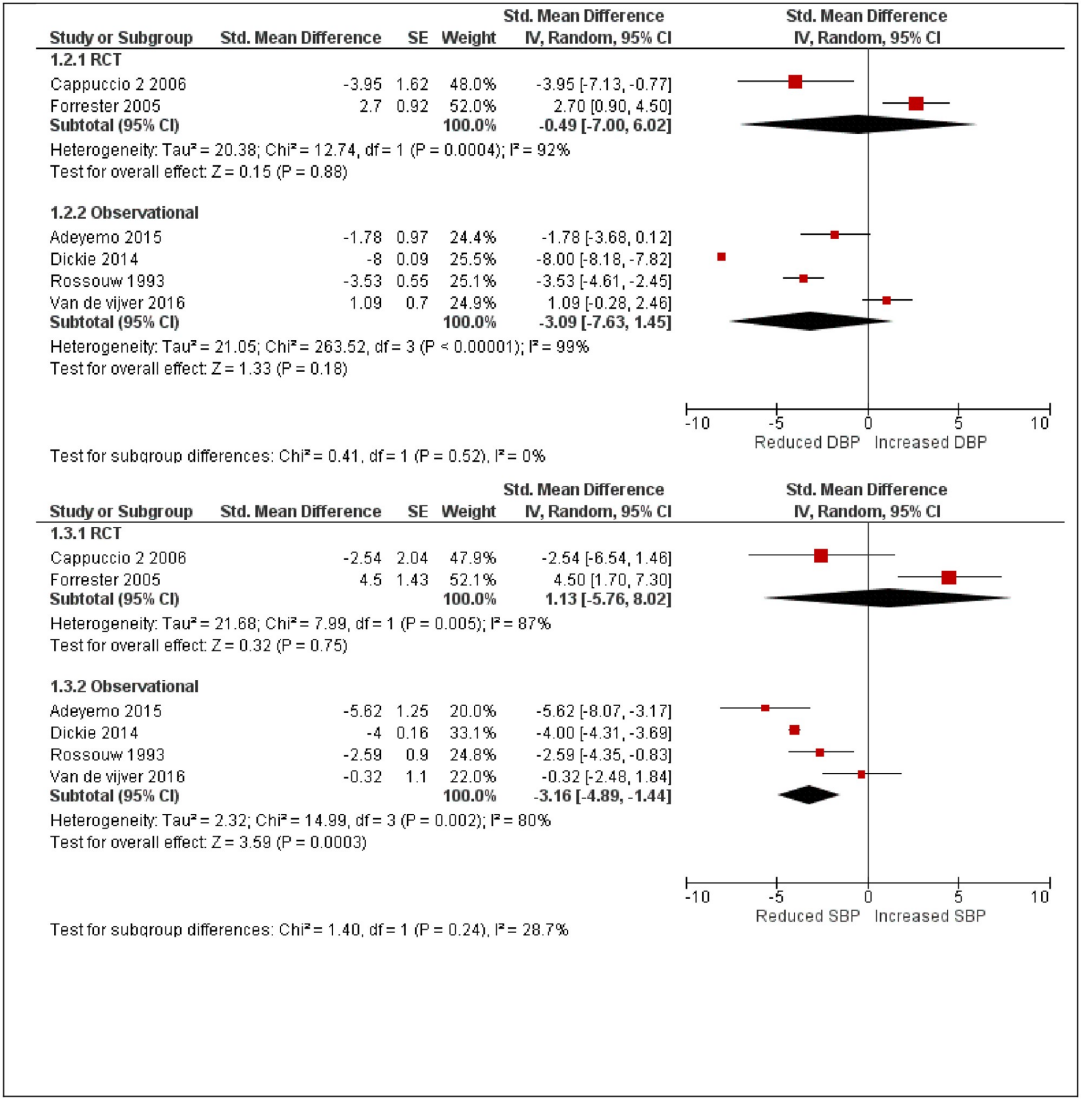
Forest plot showing changes in blood pressure by study design.

Fig 2 represents forest plots summarising the effects of 6 studies on systolic and diastolic blood pressure. A statistically significant overall reduction in SBP was observed, -3.3 (95% CI -4.64 to -1.96 mmHg). On the other hand, the overall reduction in DBP was -2.26 (95% CI -6.36 to 1.85 mmHg) and was not statistically significant. However, as expected there was a significant amount of heterogeneity among the studies which was statistically significant, with (I^2^ = 68%) for the pooled estimate of SBP and (I^2^ = 99%) for the DBP pooled estimate, with p <0.001 for both estimates. Hence, our results should be interpreted with caution, since the changes in blood pressure may be explained by heterogeneity and not by chance. Plots showing the change in blood pressure by study design can be seen in Fig 3.

#### Sources of heterogeneity

Table 2 summarises the results of a meta-regression analysis conducted in Robumeta to explore sources of heterogeneity. Study design and Intervention type were explored since they were thought to account for most of the heterogeneity observed in the meta-analysis. Study design and Intervention used were observed to account for 100% of the variation (R^2^ = 100%), for both systolic and diastolic blood pressure respectively. The RCT study designs were observed to increase SBP by 5.07mmHg (95% CI 0.82mmHG to 9.32mmHg) compared to observational studies, while quasi-experimental studies showed no significant change in SBP compared to observational studies -2.27mmHg (95%CI -5.05mmHg to 0.51mmHg). Also, a physical activity intervention reduced SBP by -3.68mmHg (95%CI -5.86mmHg to -1.50mmHg) compared to health promotion interventions, while salt interventions reduced SBP by -3.03mmHg (95%CI -6.05mmHg to -0.20mmHg) compared to health promotion interventions. The Anova test of moderators was significant with p<0.001. For DBP, quasi-experimental studies led to a significant reduction in DBP -4.61mmHg (95%CI -6.36mmHg to -2.87mmHg) compared to observational studies, while RCT resulted in a reduction of -2.23mmHg (95%CI -5.15mmHg to 0.70mmHg). Similarly, a physical activity intervention reduced DBP by -9.10mmHg (95%CI -10.47mmHg to -7.71mmHg) compared to health promotion interventions, with salt interventions increasing DBP by 1.75mmHg (95%CI -0.43mmHg to 3.93mmHg) compared to health promotion interventions. However, the statistical power for testing heterogeneity may not be sufficient because only six papers were included in this meta-analysis and regression.

**Table 2:** Meta-regression analysis of study characteristics on average net reduction in blood pressure.

SBP (R^2^ = 100%)						DBP (R^2^ = 100%)				
Characteristics	Estimate	SE	P value	LCI	UCI	Estimate	SE	P value	LCI	UCI
**Intercept**	-0.32	1.10	0.77	-2.48	1.84	1.09	0.7	0.12	-0.28	2.46
**Quasi-experimental study**	-2.27	1.42	0.11	-5.05	0.51	-4.61	0.9	<0.001	-6.36	-2.87
**RCT**	5.07	2.17	0.02	0.82	9.32	-2.23	1.5	0.14	-5.15	0.70
**Physical activity intervention**	-3.68	1.11	0.009	-5.86	-1.50	-9.10	0.7	<0.001	-10.47	-7.71
**Salt intervention**	-3.03	1.53	0.04	-6.05	-0.02	1.75	1.1	0.12	-0.43	3.93

### Publication bias

**Fig 4:**
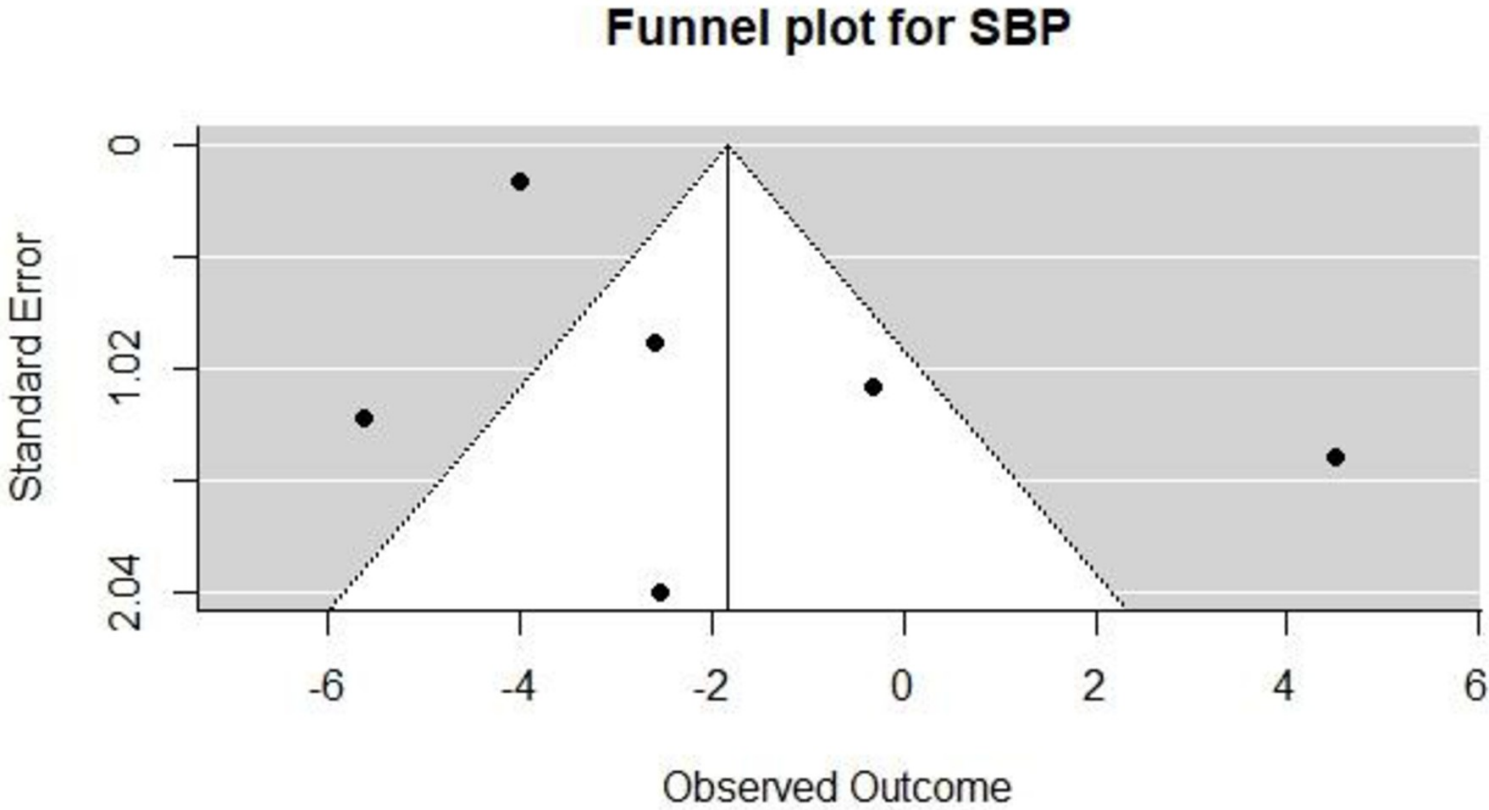
Funnel plot for SBP.

**Fig 5:**
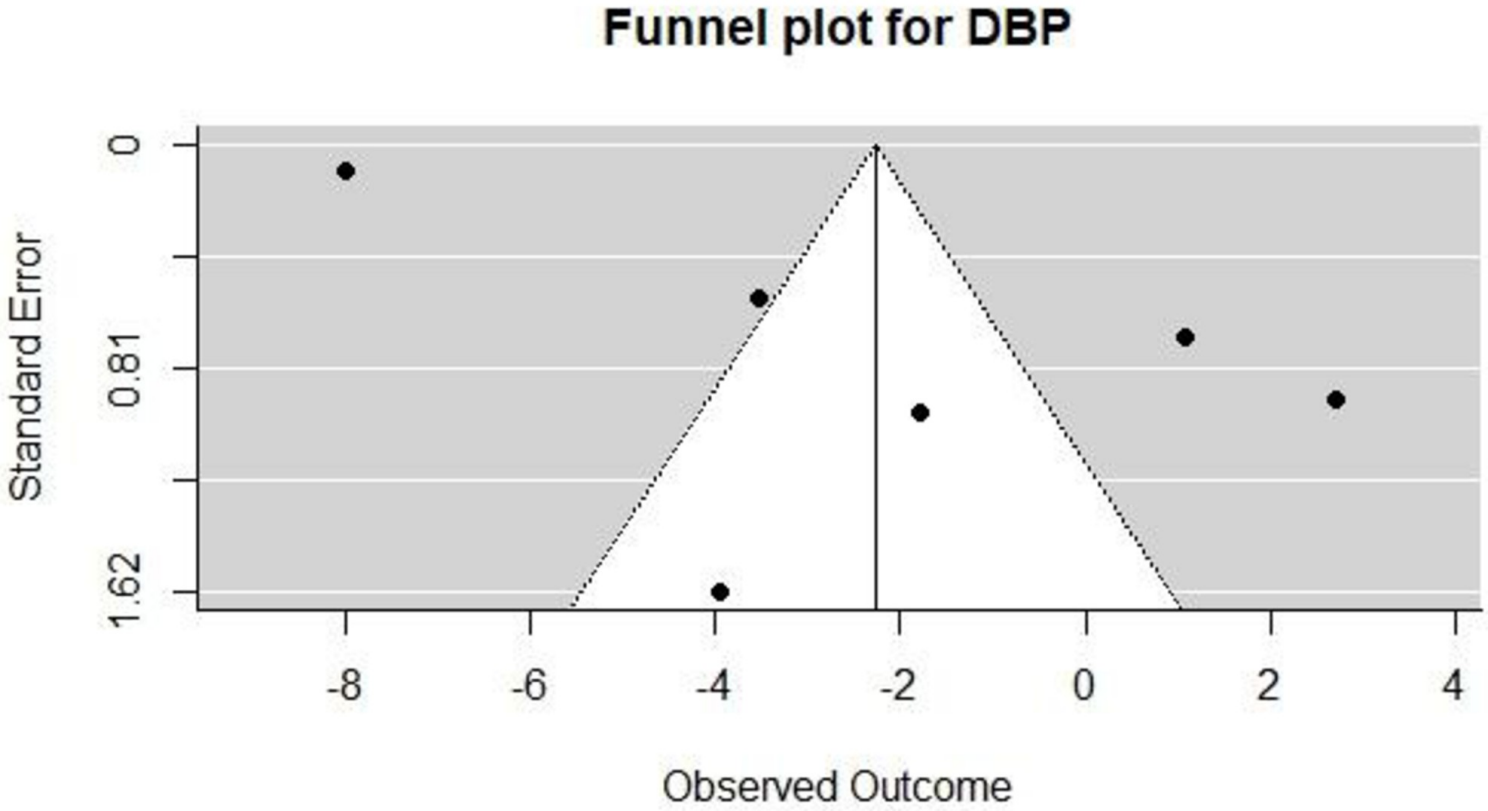
Funnel plot for DBP.

We created funnel plots by plotting the treatment effect against the standard error of the treatment effect (see Figs 4 and 5). For DBP and SBP, the funnel plot was symmetrical around the mean effect size line. Also, Egger’s test for funnel plot asymmetry was calculated, with p = 0.27 for SBP and 0.02 for DBP; this suggests the presence of bias in the DBP plot since 0.02 is statistically significant. However, we assessed for funnel plot asymmetry using less than the recommended 10 studies necessary to give the tests sufficient power. Hence, these findings may not be accurate [39].

## Discussion

### Salt limiting interventions

Our review and meta-analysis show that interventions aimed at limiting salt intake reduced the intake of salt leading to a decrease in SBP, DBP or both. Also, the meta-regression showed they were more effective in reducing blood pressure than health promotion interventions. Decreases in SBP varied from 4 mmHg to 5.6mmHg, while DBP changed by 1.02mmHg. Also, the changes in blood pressure were associated with a decrease in urinary sodium excretion of between 25mmol/l/24 hour to 52mmol/l/24 hour, which is equivalent to approximately 1.5g to 3g of salt intake daily. Our results are consistent with several meta-analyses. For example, an analysis of 78 trials [40] to determine whether reductions in blood pressure achieved in dietary salt reduction trials was consistent with estimates derived from blood pressure and sodium intake in measurements from different populations; found that a reduction in sodium intake by about 50mmol/l/day (equivalent to 3g) was associated with a 5mmHg decrease in SBP, and 2.0mmHg decrease in DBP. Furthermore, a salt reduction campaign carried out in the UK in 2003 [41], led to a 15% reduction in salt intake (p<0.05) [42] with 6000 fewer deaths from cardiovascular disease. The campaign was also found to be cost-effective, saving the UK government an estimated 1.5 billion pounds a year [43], and has been copied by several nations [44, 45]. However, two recent papers claimed that, firstly, a lower salt intake was associated with higher cardiovascular mortality [46] and, secondly, there was a J-shaped association between salt intake and cardiovascular risk [47]. It should be noted that these papers have many methodological flaws, such as measurement error in assessing daily salt intake, confounding factors not controlled for, and reverse causality. Many countries are adopting a policy of limiting salt intake, the challenge now is encouraging low income countries to adopt this policy.

### Health promotion interventions

We observed conflicting results on the effect of mass media campaigns with or without individual counselling on lifestyle modifications and hypertension. With some studies finding significant reductions in SBP, DBP, or hypertension prevalence and others demonstrating no change or negative results. However, it is important to mention that there was significant heterogeneity in study design and target population among the studies, as well as a significant risk of bias from the poor-quality studies. Nevertheless, health promotion interventions, many of which include salt limiting components, have been implemented by several countries with successful results, for example, an evaluation of one such program implemented in Indonesia and India reported a blood pressure decline of 1-3mmHg in India, and 7-16mmHg in Indonesia [48]. A similar intervention was implemented in a workplace and rural communities in China [49]. Over the 9-year period of the study, greater declines in smoking and alcohol intake levels, and blood pressure (SBP/DBP of −1.4/0.5 mmHg in men and −3.4/−1.0 mmHg in women, p< 0.001) were seen in the intervention compared to the control villages. Also, an increase in the intake of fruits and vegetables was observed, which is similar to what was observed in our review [49].

Finally, the use of food pricing and community resources such as community pharmacies were other strategies we uncovered, which can also be effective tools in carrying out health promotion, education and screening for high-risk individuals. However, there was paucity of evidence from our review to fully support the use of these interventions.

### Physical activity

In addition, policies aimed at increasing physical activity showed some promise. Even though we only identified one study which reported an isolated decrease in DBP [34]. A similar study by *Mcdonnell et al* [50] observed a decrease in DBP in individuals aged 30 and below in both males and females. Although, *Dickie et al* [34] *only* recruited females, both studies had similar age distributions. However, because levels of physical activity were not assessed during the follow-up period, it cannot be concluded that physical activity is associated with a decrease in blood pressure.

### Strengths

We chose to adopt a broad scope for our review question as opposed to a narrow one since we wanted a comprehensive synthesis of evidence to inform public health policy and practice in SSA and there was anticipation of sparse evidence. However, broad review questions are more prone to heterogeneity, are more difficult to interpret, and may not be generalisable to other settings or populations [51].

Four databases were searched, more than the recommended three databases recommended by the Cochrane handbook. Also, we searched Grey literature, study references and contacted authors for missing data, all to minimise bias. Even though we did not search all available databases, we believe our search strategy was robust enough to minimise the number of articles missed. Also, we did not limit our search by time or language which further limits the impact of publication bias.

Furthermore, we were able to conduct a meta-analysis and explain sources of heterogeneity using a meta-regression which similar studies conducted in Sub-Saharan Africa were unable to accomplish.

### Limitations

We were only able to include 13 studies in our review, representing 6 out of 46 countries; which may not be fully representative of the region and may only reflect changes in the countries represented. Also, the number of studies limits the power of our study. Most of our studies were either judged to be of low or fair quality and at risk of bias. Study screening, data extraction and quality assessment were completed by one reviewer and cross-checked by a second reviewer. Nevertheless, the study could still be liable to reviewer bias. In addition, we were unable to do a sub-group analysis exploring the effect of follow-up time, location and sample size on the outcomes due to an inadequate number of studies. Even though we retrieved studies published in different languages, they did not meet our inclusion criteria. Hence, our findings may not represent countries where English is not spoken. Also, our research is subject to moderate to high levels of heterogeneity due to the interventions and study designs used in the studies we assessed, as demonstrated in the meta-regression analysis.

## Conclusion

### Public health implications

The systematic review and meta-analysis in this report suggest that the rising burden of hypertension in SSA with sparse, effective population-wide interventions remains a huge public health concern in the region. We observed that a variety of community/population health promotion interventions were commonly used. Several studies also sought to pilot or establish the feasibility of salt limiting interventions. We also reported on strategies that could be effective in high-risk individuals. Our findings suggest that salt limiting and health promotion interventions can be effective in modifying risk factors of hypertension, and by extension reducing blood pressure. However, our findings must be interpreted with caution due to the limitations previously mentioned. The majority of salt limiting interventions we identified were achieved through health education; however, evidence suggests that comprehensive public health programmes which combine either food reformulation, food labelling, food subsidies, or taxation with health promotion/education, have been more successful than ones that employed only consumer-led interventions (e.g. health education), and can be implemented in SSA. However, several of the studies included in our review were at risk of bias.

### Recommendations for future research

We recommend that more population-wide, high quality, representative studies need to be conducted in more SSA countries to explore the effectiveness of the interventions we described and to better inform public health policy and practice. Also, study outcomes need to be reported in formats that can easily be extracted for meta-analyses. Also, should more evidence become available, a meta-analysis will be very useful in providing a point estimate of the interventions being used.

## Supporting information

S1 Checklist Prisma checklist. (DOC)

S1 Appendix Ovid/Embase search strategy. (DOCX)

S2 Appendix Cochrane and WES search strategy. (DOCX)

S1 File Risk of bias observational studies. (DOCX)

S2 File Risk of bias RCT. (DOCX)
