# Supplementary material for: The impact of interventions for the primary prevention of hypertension in Sub-Saharan Africa: A systematic review and meta-analysis
Source: PLoS One. 2019 Jul 19;14(7):e0219623. doi: 10.1371/journal.pone.0219623 (PMC6641142; doi:10.1371/journal.pone.0219623)
Supplement: S2 Appendix — (DOCX) [file pone.0219623.s003.docx]

**S2 Appendix. Cochrane and WES search strategy**

***Cochrane search***

| ID | Search |  |
| --- | --- | --- |
| #1 | hyper*tens* or high blood pressure: ti,ab,kw (Word variations have been searched) |  |
| #2 | MeSH descriptor: [Hypertension] explode all trees | |
| #3 | (primary or population) prevention | |
| #4 | MeSH descriptor: [Primary Prevention] explode all trees |  |
| #5 | dietary salt or sodium chloride |  |
| #6 | MeSH descriptor: [Sodium Chloride, Dietary] explode all trees |  |
| #7 | sub*sahara* africa or ssa or third world country or developing country or low income countr* |  |
| #8 | MeSH descriptor: [Africa South of the Sahara] explode all trees |  |

**Web of Science search terms**

| #6 #5 AND #4 AND #1 |
| --- |
| DocType=All document types; Language=All languages; |
| #5 #3 OR #2 |
| DocType=All document types; Language=All languages; |
| #4 TS= (sub*sahara* africa) |
| DocType=All document types; Language=All languages; |
| #3 TS= (dietary salt) OR TS=(sodium chloride) |
| DocType=All document types; Language=All languages; |
| #2 TS= (primary prevention) OR TS= (population prevention) OR TS= (primary intervention) |
| DocType=All document types; Language=All languages; |
| #1 TS= (hyper*tens*) OR TS=(high blood pressure) |
| DocType=All document types; Language=All languages; |
