# Supplementary material for: The impact of interventions for the primary prevention of hypertension in Sub-Saharan Africa: A systematic review and meta-analysis
Source: PLoS One. 2019 Jul 19;14(7):e0219623. doi: 10.1371/journal.pone.0219623 (PMC6641142; doi:10.1371/journal.pone.0219623)
Supplement: S1 File — (DOCX) [file pone.0219623.s004.docx]

**S1 file. Risk of bias observational studies**

|  | The National Heart, Lung, and Blood Institute (NHLBI) study quality assessment tool for observational studies | | | | | | |
| --- | --- | --- | --- | --- | --- | --- | --- |
|  |  | First Author | | | | | |
|  | Item | Ruopeng An, 2013 | Gary Dowse, 1995 | Henry Tagoe, 2011 | Edelweiss Wentzel-Viljoen, 2017 | Steven Van de Vijver, 2016 | Kasha Dickie, 2014 |
| 1 | Study question | 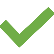 | 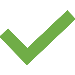 | 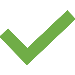 | 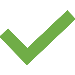 | 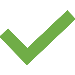 | 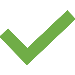 |
| 2 | Study population selection | 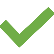 | 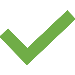 | 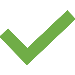 | 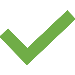 | 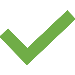 | 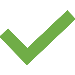 |
| 3 | Eligible population participation | 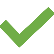 | 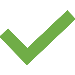 | 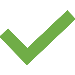 | 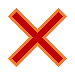 | 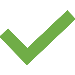 | 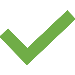 |
| 4 | Uniform eligibility criteria and selection | 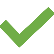 | 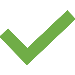 | 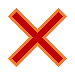 | 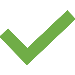 | 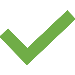 | 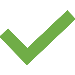 |
| 5 | Sample size justification | 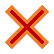 | 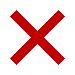 | 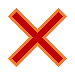 | 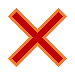 | 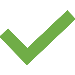 | 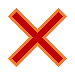 |
| 6 | Exposure assessment prior to outcome measurement | 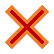 | 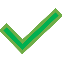 | 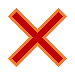 | 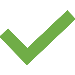 | 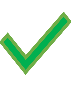 | 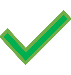 |
| 7 | Sufficient timeframe for effect | 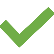 | 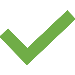 | 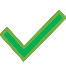 | 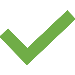 | 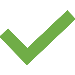 | 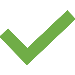 |
| 8 | Different levels of exposure of interest | 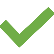 | 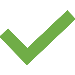 | 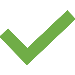 | 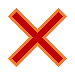 | 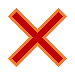 | 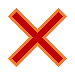 |
| 9 | Exposure measures and assessment | 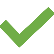 | 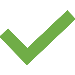 | 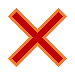 | 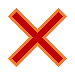 | 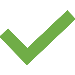 | 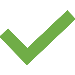 |
| 10 | Repeated exposure assessment | 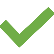 | 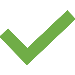 | 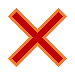 | 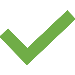 | 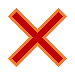 | 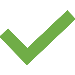 |
| 11 | Outcome measures | 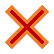 | 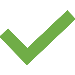 | 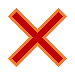 | 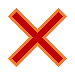 | 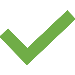 | 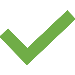 |
| 12 | Blinding of outcome assessors | NA | NA | NA | NA | NA | NA |
| 13 | Follow-up rate | NA | 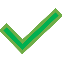 | NA | NA | 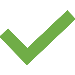 | 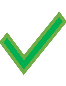 |
| 14 | Statistical analyses | 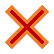 | 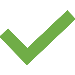 | 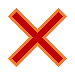 | 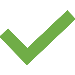 | 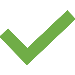 | 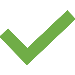 |
|  | Quality rating  Good= low risk of bias  Fair= fair risk of bias  Low= high risk of bias | fair | good | low | fair | good | fair |

Quasi-experimental studies

|  | The National Heart, Lung, and Blood Institute (NHLBI) study quality assessment tool for Pre-Post studies with no control group (Quasi-experimental design) | | | | | | |
| --- | --- | --- | --- | --- | --- | --- | --- |
|  | First Author | | | | | | |
|  | Item | J.P Mtabaji, 1990 | | Thandi Pouane, 2006 | Afia Marfo, 2016 | Adebowale Adeyemo, 2015 | Jacques Rossouw, 1993 |
| 1 | Study question | | 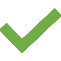 | 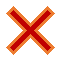 | 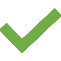 | 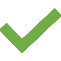 | 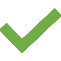 |
| 2 | Eligibility criteria and study population | | 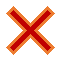 | 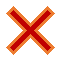 | 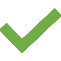 | 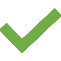 | 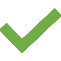 |
| 3 | Study participants representative of population | | 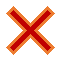 | 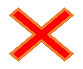 | 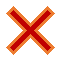 | 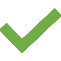 | 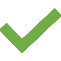 |
| 4 | Enrolment of eligible participants | | 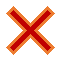 | 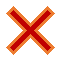 | 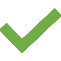 | 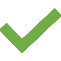 | 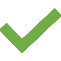 |
| 5 | Sample size | | 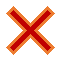 | 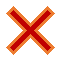 | 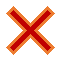 | 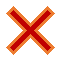 | 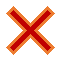 |
| 6 | Description of intervention | |  |  |  |  |  |
| 7 | Description, validity, and reliability of outcome measures | |  |  |  |  |  |
| 8 | Blinding of outcome assessors | |  |  |  |  |  |
| 9 | Follow-up rate | |  |  |  |  |  |
| 10 | Statistical analysis | |  |  |  |  |  |
| 11 | Multiple outcome measures | |  |  |  |  |  |
| 12 | Group-level interventions and individual-level outcome efforts | | NA | NA | NA | NA | NA |
|  | Quality rating | | low | low | fair | good | good |
