# Supplementary material for: The impact of interventions for the primary prevention of hypertension in Sub-Saharan Africa: A systematic review and meta-analysis
Source: PLoS One. 2019 Jul 19;14(7):e0219623. doi: 10.1371/journal.pone.0219623 (PMC6641142; doi:10.1371/journal.pone.0219623)
Supplement: S2 File — (DOCX) [file pone.0219623.s005.docx]

|  | **Cochrane risk of bias tool for quality assessment of randomised controlled trials** | | |
| --- | --- | --- | --- |
|  | **First Author** | | |
|  | **Item** | Francesco Cappuccio ,2006 | T. Forrester, 2005 |
| 1 | Risk of bias arising from randomisation process | 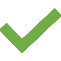 | 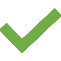 |
| 2 | Risk of bias due to deviation from intended intervention | 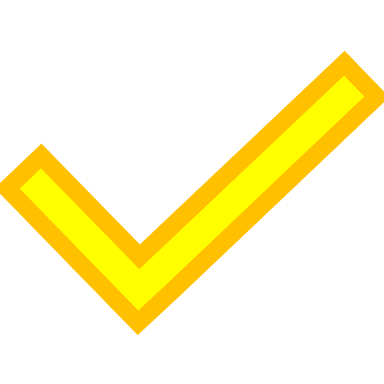 | 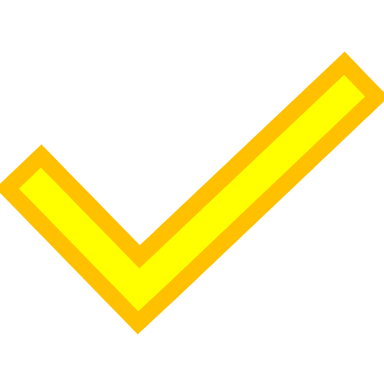 |
| 3 | Risk of bias due to missing outcome data | 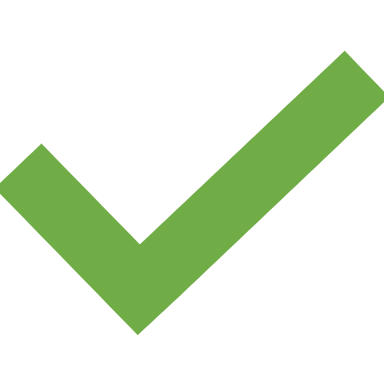 | 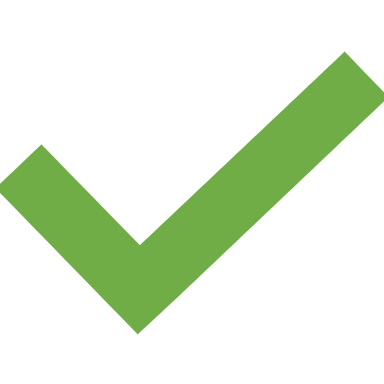 |
| 4 | Risk of bias in measurement of outcome | 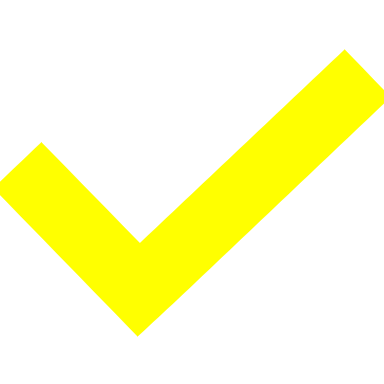 | 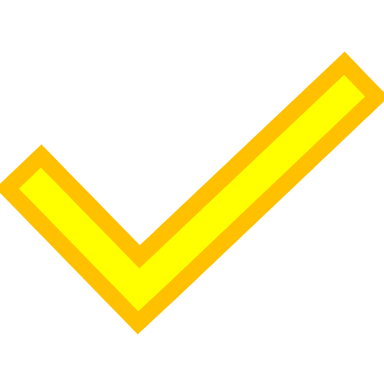 |
| 5 | Risk of bias in selection of the reported result | 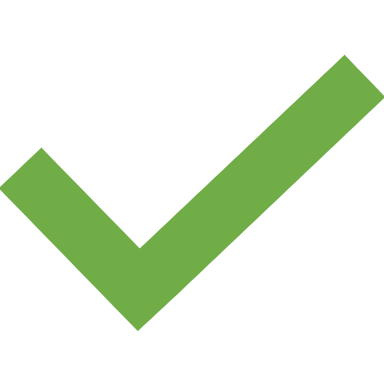 | 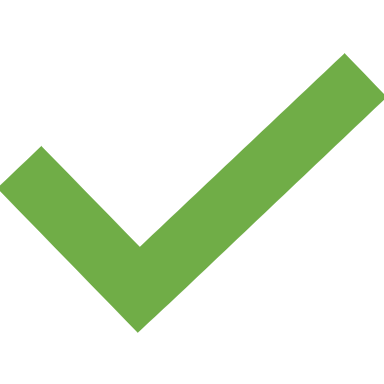 |
|  | Overall Risk of bias | 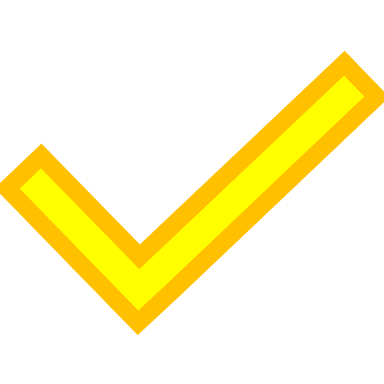 | 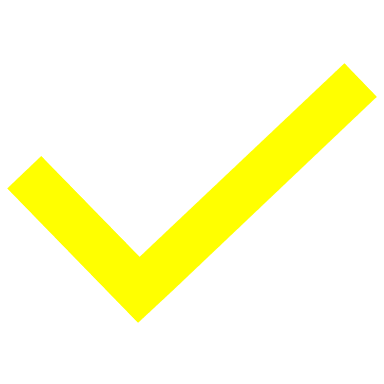 |
|  | Yellow tick = some concerns |  |  |
|  | Green tick= low risk of bias |  |  |

**S2 file. Risk of bias RCT**
